# Chagas disease and mobility: comments on the challenges for access and the right to health for migrants

**DOI:** 10.1590/0074-02760210151chgsa

**Published:** 2022-07-08

**Authors:** Paulo Cesar Peiter

**Affiliations:** 1 Fundação Oswaldo Cruz-Fiocruz

Human mobility has a rich literature in social sciences and demography and recently this theme has been approached from the perspective of immigrants’ rights, interculturality, public security, border health, disease diffusion, mental health and access to public health services.^1^ ^,^ ^2^

The article “Population movements, borders, and Chagas disease” has the merit of addressing the issue of human mobility by relating it to a neglected disease such as Chagas disease.

This discussion is also opportune as the migration issue emerges as a problem charged with misconceptions and prejudices, considering migrants a burden for public health, as well as international borders are regarded as dangerous places and security is reinforced in these regions. Nevertheless, scientific evidence shows that in fact migrants are an asset to host countries and borders create opportunities to international cooperation and to promote cultural and social diversity.

On the other hand, the UCL-Lancet Commission on Migration and Health (2018) points out that according to International Conventions on Migration signatory countries should provide to migrant’s access to the best healthcare services available.^1^

In this context the article in focus raises an important question: What are the challenges that should be faced in the intersection between Chagas and human mobility to fill the gaps between migrants and health systems achieving a better care to affected people?

The authors advocate a holistic, interdisciplinary and multisectoral approach to Chagas disease to account for its complexity and claim that epidemiology, tropical medicine, and social sciences should get together to include affected people’s subjectivities in the formulation of public policies for prevention, surveillance and control of Chagas disease, in addition to the active social participation.

According to them it is essential for research and policy building on Chagas disease that it be understood as a “health-disease-prevention-care process”, considering the perceptions and experiences of affected people, and contemplating the particularities of the territories, as well as the role of inequities in different contexts.

Based on this new approach the complexity of human mobility comes to the surface, showing a range of situations that go from daily commuting movements to international migratory movements, as well as a diversity of migrant status such as multinationals CEO’s, scholarship students, unemployed workers and several types of refugees.^1^

Each category of mobility and stage of the migration process (departure, transit and arrival to final destination) implies different risk exposures and degrees of health vulnerabilities and in turn express the inequities and varying degrees of inclusion and exclusion of migrants in host countries.

This statement is reinforced by the vulnerable condition of the people affected by Chagas disease who is subject of stigmatisation, resulting in the distancing of these people from health services. The irregular situation of many migrants further contributes to this distancing and even invisibility, for fear of sanctions and deportation, making it difficult to detect Chagas cases and consequently take care of them.

Furthermore, the various barriers that arise for migrants to access health services, the authors invite us to reflect on the possibilities of ensuring equitable access to health services for people living with Chagas disease in a transnational context.

The article proposes to use of information and communication strategies in health to enhance knowledge of people affected and health professional, especially in primary care, that is, closer to people creating bonds of trust so that the follow-up and adherence to treatments is maximised.

They also suggest that Chagas community representatives should participate in the formulation of the action strategies as they can bring their own experiences with the disease.

In conclusion, this article aligns the knowledge and perspectives of construction of knowledge and society’s responses to Chagas disease to the most advanced tendencies in science that are interdisciplinarity and social participation in the construction of sustainable and equitable solutions.

Comments on the article: Avaria A, Ventura-Garcia L, Sanmartino M, Van der Laat C. Population movements, borders, and Chagas disease. Mem Inst Oswaldo Cruz. 2022; 117: e210151.
